# Frontal White Matter Changes and Craving Recovery in Inpatients With Heroin Use Disorder

**DOI:** 10.1001/jamanetworkopen.2024.51678

**Published:** 2024-12-18

**Authors:** Pierre-Olivier Gaudreault, Sarah G. King, Yuefeng Huang, Ahmet O. Ceceli, Greg Kronberg, Nelly Alia-Klein, Rita Z. Goldstein

**Affiliations:** 1 Departments of Psychiatry and Neuroscience, Icahn School of Medicine at Mount Sinai, New York City, New York

## Abstract

**Importance:**

Amidst an unprecedented opioid epidemic, identifying neurobiological correlates of change with medication-assisted treatment of heroin use disorder is imperative. White matter impairments in individuals with heroin use disorder (HUD) have been associated with drug craving, a reliable predictor of treatment outcomes; however, little is known about structural connectivity changes with inpatient treatment and abstinence in individuals with HUD.

**Objective:**

To assess white matter microstructure and associations with drug craving changes with inpatient treatment in individuals with HUD (effects of time and rescan compared with controls).

**Design, Setting, and Participants:**

This cohort study conducted from December 2020 to September 2022 included individuals recruited from urban inpatient treatment facilities treating HUD and surrounding communities in New York City. Participants with HUD were receiving medication-assisted treatment. Data were analyzed from October 2022 to March 2023.

**Intervention:**

Between scans, inpatient individuals with HUD continued treatment and related clinical interventions. Control participants were scanned at similar time intervals.

**Main Outcomes and Measures:**

Changes in white matter diffusion metrics (fractional anisotropy and mean, axial, and radial diffusivities) assessed voxelwise with general linear models in addition to baseline and cue-induced drug craving, and other clinical outcome variables (mood, sleep, affect, perceived stress, and therapy attendance).

**Results:**

Thirty-four individuals with HUD (mean [SD] age, 40.5 [11.0] years; 9 women [36%]; 3 Black [9%], 17 White [50%], 14 other race or ethnicity [41%]) and 25 control (mean [SD] age, 42.1 [9.0]; 7 women [21%]; 8 Black [32%], 10 White [40%], 7 other race or ethnicity [28%]) were included. Main voxelwise findings showed HUD-specific white matter microstructure changes (1 − *P* > .949), including increased fractional anisotropy and decreased mean and radial diffusivities, encompassing mostly frontal major callosal, projection, and association tracts. The increased fractional anisotropy (*r* = −0.72, *P* < .001, slope SE = 9.0 × 10^−4^) and decreased mean diffusivity (*r* = 0.69, *P* < .001, slope SE = 1.25 × 10^−6^) and/or radial diffusivity (*r* = 0.67, *P* < .001, slope SE = 1.16 × 10^−6^) in the genu and body of the corpus callosum and left anterior corona radiata in individuals with HUD correlated with a reduction in baseline craving (voxelwise 1 − *P* > .949). No other white matter correlations with outcome variables reached significance.

**Conclusions and Relevance:**

This cohort study of inpatients with HUD on medication-assisted treatment found whole-brain normalization of structural connectivity in frontal white matter pathways implicated in emotional regulation and top-down executive control. Observed associations with decreases in baseline craving further support the possibility of recovery, highlighting the relevance of these white matter markers to a major symptom of addiction, with implications for clinical outcome monitoring.

## Key Points

**Question:**

Does white matter microstructure change during medication-assisted treatment in individuals with heroin use disorder (HUD)?

**Findings:**

In this cohort study of 34 inpatient individuals with HUD and 25 healthy controls, changes in white matter microstructure were associated with treatment in the individuals with HUD cohort, characterized by increased anisotropy and decreased diffusivity in fronto-striatal white matter pathways, which correlated with decreases in baseline drug craving.

**Meaning:**

These results suggest that frontal white matter changes and associated drug craving decreases are associated with brain-behavior recovery with inpatient treatment in individuals with HUD, potentially contributing to reduced drug use and sustained abstinence.

## Teaser

This cohort study examines the association of changes in white matter microstructure in a cohort of US individuals with heroin use disorder undergoing inpatient treatment with changes in drug cravings.

## Introduction

North America is currently experiencing an opioid epidemic that caused approximately 107 000 fatal overdoses in 2021,^1^ with opioid use disorders associated with particularly high relapse rates (eg, 91%^2^ as compared with 40% to 60% in other substance use disorders).^3^ The complexity in treating heroin use disorder (HUD) stems in part from drug-related craving, the intense subjective urge to use drugs,^4^ a reliable predictor of treatment outcomes (inclusive of sustained abstinence vs propensity to relapse).^5,6^ Identifying neurobiological correlates of change during treatment, including in craving reductions, is therefore a priority.

Studies investigating neurobiological mechanisms underlying drug craving and other treatment outcomes show specific impairments in the cortico-striatal and mesolimbic pathways and neuronal projections reaching prefrontal regions.^7,8^ However, these studies have mostly relied on magnetic resonance imaging (MRI)-based functional neuroimaging-derived task-based regional changes^9,10,11^ and whole-brain functional connectivity,^12,13^ with less emphasis on structural connectivity underlying clinical symptoms. In 2023, we reported widespread whole-brain white matter impairments in individuals with cocaine and heroin use disorders as evidenced by decreased fractional anisotropy, a measure of white matter organizational coherence, and increased mean, axial, and radial diffusivities, measures associated with axonal damage and demyelination, as compared with healthy control participants.^14^ Across all participants with substance use disorders, these white matter deficits were associated with longer duration of regular use, suggestive of a cumulative and/or persistent effect, and with higher state-like (baseline) craving measures, highlighting the importance of current symptom severity.^14^ While this latter study added to the growing evidence showing whole-brain white matter deficits in individuals with HUD,^15,16,17,18,19^ far less research has assessed potential changes during abstinence or treatment, especially as associated with parallel putative changes in symptoms including craving.

A 2022 systematic review^20^ of longitudinal structural (gray and white matter) and functional (including electroencephalogram, function MRI [fMRI], and neurochemical) neuroimaging studies suggested changes consistent with recovery in abstinence across most substance use disorders, with the onset of structural changes preceding neurochemical and functional ones. Specifically in HUD (based on 20 studies), the earliest evidence for initial white matter change compared with control participants was provided in a preliminary report showing no-longer-detectable decreased fractional anisotropy in the frontal and cingulate gyri with 1 month of abstinence as compared to baseline (3 days of abstinence).^21^ Consistently, recent atlas-based studies using group-averaged tractography masks reported increased fractional anisotropy in cortico-striatal white matter tracts from approximately 2 to 8 months of abstinence in individuals with HUD, as associated with decreased drug craving scores at follow-up^22^ and predictive of longitudinal craving changes.^23^ However, these studies focused on individuals with HUD treated only with psychoeducational and social and/or physical rehabilitation, and therefore less is known about the effect of the US system of standard-of-care medication-assisted treatment (MAT) on white matter microarchitecture.

The aim of this study was to assess white matter changes with inpatient MAT in individuals with HUD as compared with control participants. Using a tensor-based, voxelwise approach to evaluate whole-brain changes in white matter, we expected normalization of diffusion metrics including increased fractional anisotropy and decreased mean, axial, and radial diffusivities with more time in treatment (approximately 14-week interval) in individuals with HUD, and minimal changes in control participants over an equivalent interval. Because different craving measures provide distinct indicators of treatment outcomes,^6^ we tested the association of white matter changes with changes in both baseline (assessed without cue exposure) and cue-induced and/or dynamic craving. A secondary objective included testing associations between these white matter changes and select behavioral clinical measures including mood, sleep, affect, perceived stress, and therapy attendance.

## Methods

### Participants

Participants undergoing inpatient MAT for HUD (30 participants treated with methadone, 4 with buprenorphine) were recruited from a substance use disorder rehabilitation facility in Queens in New York City, where they attended courses on relapse prevention, “Seeking Safety” therapy, and anger management. Twenty-one individuals with HUD in this cohort were included in our recent cross-sectional report.^14^ Healthy control participants, who did not differ statistically from HUD in age or self-reported race (Black or African American, White, other [American Indian, Asian, 2 or more races, none reported]) or gender, were recruited through advertisements and word-of-mouth from the same communities. All individuals with HUD and control participants were recruited as part of an ongoing clinical trial (NCT04112186) evaluating therapy-specific neuroimaging changes in HUD (for intervention-related outcomes to be reported after trial completion, see eMethods in Supplement 1) and completed both baseline and follow-up MRI assessments (mean [SD] time between scans, 13.9 [5.8] weeks). All individuals with HUD met *Diagnostic and Statistical Manual of Mental Disorders* (Fifth Edition) criteria for opioid use disorder (with heroin as their primary drug of choice or reason for treatment) and were stabilized on MAT (confirmed via urine toxicology in both sessions). Exclusion criteria are described in the eMethods in Supplement 1, along with data on psychiatric comorbidities. All comorbidities in individuals with HUD were in partial or sustained remission, and control participants had no current or prior substance use disorder or other psychiatric conditions. Study procedures were approved by the institutional review board of the Icahn School of Medicine at Mount Sinai, and all participants provided written informed consent. This study followed the Strengthening the Reporting of Observational Studies in Epidemiology (STROBE) reporting guidelines for cohort studies.

### Clinical Assessments

During a screening session administered prior to MRI data collection, trained clinical coordinators supervised by clinical psychologists conducted diagnostic evaluations including the Mini International Neuropsychiatric Interview 7th edition^24^ and the Addiction Severity Index.^25^ Withdrawal symptoms were assessed with the Short Opiate Withdrawal Scale^26^ and self-reported subjective heroin craving with the Heroin Craving Questionnaire (a modified version of the Cocaine Craving Questionnaire).^27,28^ Heroin dependence severity was assessed with the Severity of Dependence Scale^29^ and nicotine dependence was assessed with the Fagerström Test for Nicotine Dependence.^30^ Behavioral measures at both imaging time points in individuals with HUD and between-scan comparisons were recorded for depression and anxiety symptoms, sleep quantity and quality the night before the scan, positive and negative affect, and perceived stress (eMethods in Supplement 1). Additionally, assessments of baseline drug craving were conducted at both MRI sessions at the start of a drug cue reactivity fMRI task (“Please rate how strong your desire for heroin is currently on a scale of 0-9”), reported elsewhere.^9^ Three additional ratings after the MRI procedures assessed specific aspects of dynamic, cue-induced craving: picture-induced drug arousal (“How emotional do you feel about the picture,” assessed on a 5-point scale ranging from “calm, no emotion” to “extremely emotional”) and picture-induced drug craving (“How strong is your desire to use the substance,” assessed on a 5-point scale ranging from “no desire” to “extreme desire”) ratings of the same drug pictures used in the cue-reactivity fMRI task, and scene-induced drug craving intensity ratings in response to 34 three-second clips of a naturalistic drug-related movie presented during an fMRI task.^31^

### MRI Acquisition and Diffusion Tensor Imaging Processing

Scans were acquired using a 3.0 Tesla Skyra scanner (Siemens Healthineers AG) with a 32-channel head coil. Diffusion echo-planar sequence was acquired with opposite phase encoding along the left-right axis, monopolar diffusion encoding with 128 diffusion-weighted images (2 × 64 for each encoding phase) at single-shell maximum *b* = 1500 seconds/mm^2^, 13 reference images at *b* = 0 seconds/mm^2^, and 1.8 mm isometric voxel size (eMethods in Supplement 1). The preprocessing of the diffusion MRI data was computed according to well-established pipelines from the MRtrix3^32^ and FMRIB Software Library (FSL version 6.0)^33^ toolboxes that have been described previously including in a 2023 publication from our laboratory.^14^ Tensor-based quantitative maps of diffusion metrics (fractional anisotropy and mean, radial, and axial diffusivities) were generated and used to perform tract-based spatial statistics (TBSS) whole-brain voxelwise analyses across all participants by creating a white matter skeleton, which was registered to the Montreal Neurological Institute (MNI) standard space MNI152 template, from the averaged thresholded fractional anisotropy.^34^

### Statistical Analyses

Group comparisons for demographic, neuropsychological, and self-reported drug use measures between control participants and individuals with HUD were assessed with Student *t* tests for continuous variables and χ^2^ tests for categorical variables (Table 1). Variables that reached the familywise error (FWE) threshold of *P* < .005 (.05 across 11 statistical tests) were considered to differ significantly between the groups. Commonly used drugs (alcohol and cannabis) were also compared between the groups using the same statistical tests with a FWE threshold of *P* < .008 (.05 across 6 statistical tests). Although nicotine use was assessed, no between-group comparisons were carried out for this measure because 33 individuals with HUD and only 1 control participant reported being current smokers. Table 2 summarizes the treatment-related changes in behavioral measures and both baseline and cue-induced drug craving variables. Paired *t* tests with FWE-corrected thresholds were used to assess significant changes from baseline to follow-up MRI (*P* < .007 [.05 across 7 statistical tests] for the 7 behavioral measures and *P* < .013 [.05 across 4 statistical tests] for the 4 drug craving variables). Comparisons reaching *P* < .05 without correction are reported in this analysis.

**Table 1.** Baseline Demographic Characteristics, Neuropsychological Measures, and Drug Use Variables

Characteristics	Mean (SD)^a^		Statistical analysis	
	Control (n = 25)	Individuals with HUD (n = 34)		
			Test statistic	*P* value^b^
**Demographics**				
Age, y	40.5 (11.0)	42.1 (9.0)	t_57_ = −0.6	.54
Gender				
Men, No. (%)	16 (64)	27 (79)	χ^2^_1_ = 1.7	.19
Women, No. (%)	9 (36)	7 (21)		
Education, y	16.2 (3.3)	12.2 (1.9)	t_57_ = 5.8	<.001
Race				
Black or African American, No. (%)	8 (32)	3 (9)	χ^2^_2_ = 5.2	.08
White, No. (%)	10 (40)	17 (50)		
Other, No. (%)^c^	7 (28)	14 (41)		
**Neuropsychological measures**				
Intracranial volume, L	1.62 (0.13)	1.70 (0.15)	*t*_57_ = −2.3	.03
Neuropsychological and self-reported tests				
WRAT–Reading scale standard score	108.8 (9.3)	95.0 (12.1)	*t*_56_ = 4.7	<.001
WASI–Matrix Reasoning scaled score	12.0 (3.2)	10.5 (3.1)	*t*_56_ = 1.8	.08
Handedness				
Right, No. (%)	18 (72)	27 (79)	χ^2^_1_ = 0.4	.51
Left, No. (%)	7 (28)	7 (21)		
Baseline visit depression (BDI)^d^	3.3 (4.7)	13.8 (11.1)	*t*_57_ = −4.5	<.001
Baseline visit anxiety (BAI)^d^	2.1 (4.1)	10.6 (9.3)	*t*_57_ = −4.3	<.001
**Drug use variables**				
Regular nicotine use, current, No. (%)	1 (4)	33 (97)	NA^e^	NA^e^
Fagerström test for nicotine dependence	NA	3.6 (1.6)	NA	NA
Cigarette use during past 30 d, d	NA	28.8 (5.4)	NA	NA
Daily cigarettes use per d, No. (%)				
≤10	NA	28 (82)	NA	NA
11-20	NA	5 (15)	NA	NA
≥21	NA	1 (3)	NA	NA
Regular alcohol use, No. (%)	14 (56)	21 (62)	χ^2^_1_ = 0.2	.66
Lifetime alcohol regular use, y	12.3 (11.2)	14.5 (10.8)	*t*_32_ = −0.6	.58
Alcohol use during past 30 d, d	6.9 (8.2)	0.0 (0.0)	*t*_33_ = 3.9	<.001
Regular cannabis use, No. (%)	7 (28)	21 (62)	χ^2^_1_ = 8.7	.003
Lifetime cannabis regular use, y	3.0 (2.1)	13.0 (7.9)	*t*_26_ = −3.3	<.001
Cannabis use during past 30 d, d	0.4 (1.1)	0	*t*_26_ = 1.8	.08
Heroin use				
Age of first use, y	NA	24.2 (8.4)	NA	NA
Years of regular use	NA	10.7 (7.2)	NA	NA
Current abstinence, d	NA	176.6 (215.3)	NA	NA
Heroin use during past 30 d, d	NA	0.4 (1.2)	NA	NA
Self-reported methadone dosage, mg	NA	107.2 (57.4)^f^	NA	NA
Self-reported buprenorphine dosage, mg	NA	10.0 (9.7)^g^	NA	NA
Dependence severity, SDS score	NA	10.9 (3.6)	NA	NA
Withdrawal symptoms, SOWS score	NA	3.7 (4.9)	NA	NA
Subjective craving, HCQ score	NA	39.9 (12.6)	NA	NA
Treatment adherence				
Therapy attendance, h	NA	10.6 (4.9)	NA	NA
Time between scans, d	91.1 (50.3)	101.7 (31.7)	*t*_57_ = −1.0	.33

Abbreviations: BAI, Beck Anxiety Inventory; BDI, Beck Depression Inventory; HCQ, Heroin Craving Questionnaire; HUD, heroin use disorder; NA, not applicable; SDS, Severity of Dependence Scale; SOWS, Short Opiate Withdrawal Scale; WASI, Wechsler Abbreviated Scale Intelligence; WRAT, Wide Range Achievement Test.

^a^ Missing data for each variable: 1 data point for WASI; 1 data point for WRAT; 1 data point for years of alcohol use; 3 data points for cannabis regular use; 2 data points for years of heroin regular use.

^b^ To correct for multiple comparisons, *P* values were considered significant at *P* < .005 (0.05 across 11 statistical tests) for the demographics/neuropsychological tests and at *P* < .008 for the drug use variables (0.05 across 6 tests).

^c^ Other category includes American Indian, Asian, 2 or more races, and none reported.

^d^ Time effects on these measures in individuals with HUD are presented in Table 2.

^e^ Since only 1 healthy control participant reported currently using nicotine, no statistical tests were done to compare the groups on measures of nicotine use.

^f^ Total of 30 individuals.

^g^ Total of 4 individuals.

**Table 2.** Behavioral and Drug-Related Craving Measures Repeated After Approximately 15 Weeks of Inpatient Treatment in Individuals With Heroin Use Disorder (HUD)

Characteristics	Mean (SD) (n = 34)		Statistical analyses	
	Baseline MRI	Follow-up MRI	Test statistic	*P* value^a^
Behavioral measures				
Depression (BDI)	13.8 (11.1)	12.0 (10.7)	t_31_ = 1.7	.10
Anxiety (BAI) score	10.6 (9.3)	10.7 (10.4)	t_32_ = 0.1	.91
Sleep quantity, h	6.9 (1.7)	6.4 (1.9)	t_33_ = 1.6	.12
Sleep quality score	3.5 (1.2)	3.7 (1.2)	t_33_ = −0.5	.65
PANAS–positive score	31.3 (8.9)	34.2 (8.6)	t_32_ = −2.0	.05
PANAS–negative score	20.6 (8.9)	17.9 (6.4)	*t*_32_ = 1.9	.07
PSS score	27.9 (7.3)	26.9 (7.1)	t_32_ = 0.8	.44
Heroin use				
Current abstinence, d	176.6 (215.3)	249.3 (231.0)	t_33_ = 4.56	<.001
Past 30-d use, d	0.2 (0.7)	0.8 (2.8)	t_33_ = −1.07	.29
Craving variables				
Baseline (state-like) measure				
Pretask craving rating	3.3 (2.4)	2.0 (2.7)	t_33_ = 3.5	.001
Cue-Induced (dynamic) measures				
Drug cue ratings–arousal	2.3 (0.8)	1.9 (0.8)	t_33_ = 2.5	.02
Drug cue ratings–craving	1.9 (1.0)	1.5 (0.8)	t_33_ = 2.4	.02
Movie scene-induced craving intensity	1.3 (1.1)	0.8 (0.9)	t_31_ = 3.9	.001

Abbreviations: BAI, Beck Anxiety Inventory; BDI, Beck Depression Inventory; MRI, magnetic resonance imaging; PANAS, Positive and Negative Affect Schedule; PSS, Perceived Stress Scale.

^a^ To correct for multiple comparisons, *P* values significance was set at *P* < .007 (.05 between 7 statistical tests) for the behavioral measures and *P* < .013 (.05 between 4 statistical tests) for the drug craving variables. Missing data for each variable: 2 data points for BDI (follow-up MRI); 1 data point for BAI (follow-up MRI); 1 data point PANAS (follow-up MRI); 1 data point for PSS (follow-up MRI); 2 data points for scene-induced craving intensity (1 for baseline MRI and 1 for follow-up MRI).

Whole-brain white matter analyses were carried out using the FSL tool Randomise, a general linear model for non-parametric permutation inferences^35^ using 10 000 permutations of shuffled labels to generate the null distribution. This is a standard approach to voxelwise statistical testing of DTI metrics; hypothesis tests derived from comparison against the null distribution are robust against false-positive results that commonly arise when assumptions of normality are violated in parametric statistical tests. To investigate white matter changes, a map of between-scan change (ie, baseline MRI − follow-up MRI) for each diffusion metric was computed and used in a design matrix coding for independent groups *t* tests in accordance with the FSL user guide (2 contrasts: control greater than HUD and control less than HUD) with covariate adjustments for education, age, and intracranial volume (ICV), in line with common practices. Intracranial volume was estimated with the Freesurfer Software Suite version 7.2 (Martinos Center for Biomedical Imaging)^36^ from structural T1-weighted images. To account for multiple voxelwise comparisons, Threshold-Free Cluster Enhancement (TFCE) correction, which enhances areas of signal exhibiting spatial contiguity to better discriminate clusters, was applied.^37^ The significance threshold was set to *P* < .05 in 2-tailed tests (displayed as 1 − *P* > .949 on the statistical maps for visualization purposes). Neuroanatomical localization of white matter tracts was performed with the FSL atlasquery toolbox and the JHU ICBM-DTI-81 White-Matter Labels atlas,^38^ with an average probability of region overlap threshold of 2%.

#### Other Objectives

We tested whether the specific changes in white matter diffusion metrics observed in individuals with HUD were associated with treatment-related outcomes including craving variables, selected behavioral measures, treatment adherence, and self-reported methadone and/or buprenorphine dosages. Targeted voxelwise correlations between the change in white matter diffusion metrics and the mean-centered change values of the clinical outcome variables showing a significant change from baseline to follow-up MRI (ie, baseline craving [pretask] and cue-induced craving [scene-induced]) were performed within a mask of the significant voxels where individuals with HUD showed increased fractional anisotropy at the follow-up MRI (ie, 12 514 voxels). Although changes in picture-induced drug arousal and craving did not reach corrected significance levels, we included these measures in voxelwise correlation analyses, in addition to therapy attendance, for completeness. The correlation analyses significance threshold was set to 1 − *P* > .949 (TFCE-corrected). The magnitude of the correlations (*r*) was estimated from the extracted white matter metrics in the mask of significant voxels with IBM SPSS statistics version 27 (IBM Corp).

## Results

### Demographic, Neuropsychological, and Behavioral Measures

Thirty-four inpatient individuals with HUD (mean [SD] age, 42.1 [9.0] years; 27 men [79%]; 3 Black [9%], 17 White [50%], 14 other race or ethnicity [41%]), with and mean time on MAT of 163.5 days (range, 25-482 days), and 25 control participants (mean [SD] age, 40.5 [11.0] years; 16 men [64%]; 8 Black [32%], 10 White [40%], 7 other race or ethnicity [28%]) were recruited for this study (Table 1). The control and individuals with HUD groups did not differ significantly on days between scans and ICV, but did differ in education (mean [SD]: control, 16.2 [3.3] years vs individuals with HUD, 12.2 [1.9] years). Between-group differences were observed in verbal IQ (mean [SD] WRAT-Reading scale: individuals with HUD, 95.0 [12.1] vs controls, 108.8 [9.3]) and baseline depression (mean [SD] Beck Depression Inventory: individuals with HUD, 13.8 [11.1] vs controls, 3.3 [4.7]) and anxiety symptoms (mean [SD] Beck Anxiety Inventory: individuals with HUD, 10.6 [9.3] vs control, 2.1 [4.1]). The groups also differed on nicotine, alcohol, and cannabis use, where more individuals with HUD reported regular use of nicotine (not enough control were available for statistical comparison) and cannabis (inclusive of more years of regular cannabis use) with an opposite pattern for days of alcohol use in the past month (as expected from the controlled inpatient environment).

As expected for this inpatient population, the self-reported current abstinence duration was significantly longer at follow-up MRI compared with the baseline MRI, and there was no significant difference in self-reported heroin use in the past 30 days (mean less than 1 day at both sessions). There were significant decreases in both baseline craving and scene-induced craving intensity in the individuals with HUD at the follow-up MRI compared with the baseline (mean [SD] pretask craving, 2.0 [2.7] vs 3.3 [2.4]; mean [SD] scene-induced craving, 0.8 [0.9] vs 1.2 [1.1]). Decreases between the baseline MRI and follow up were observed for the drug picture-induced arousal (mean [SD], 2.3 [0.84] vs 1.9 [0.79]) and craving ratings (mean [SD], 1.9 [1.0] vs 1.5 [0.8]), and an increase was observed in positive affect (mean [SD] PANAS–positive, 31.3 [8.9] vs 34.2 [8.6]) with an accompanying decrease in negative affect (mean [SD] PANAS–negative, 20.6 [8.9] vs 17.9 [6.4]). There were no significant changes in the other select behavioral outcome measures (encompassing mood, sleep, and perceived stress) in the HUD group.

### Whole-Brain White Matter Changes and Correlations With Clinical Outcome Measures

**Figure 1.**
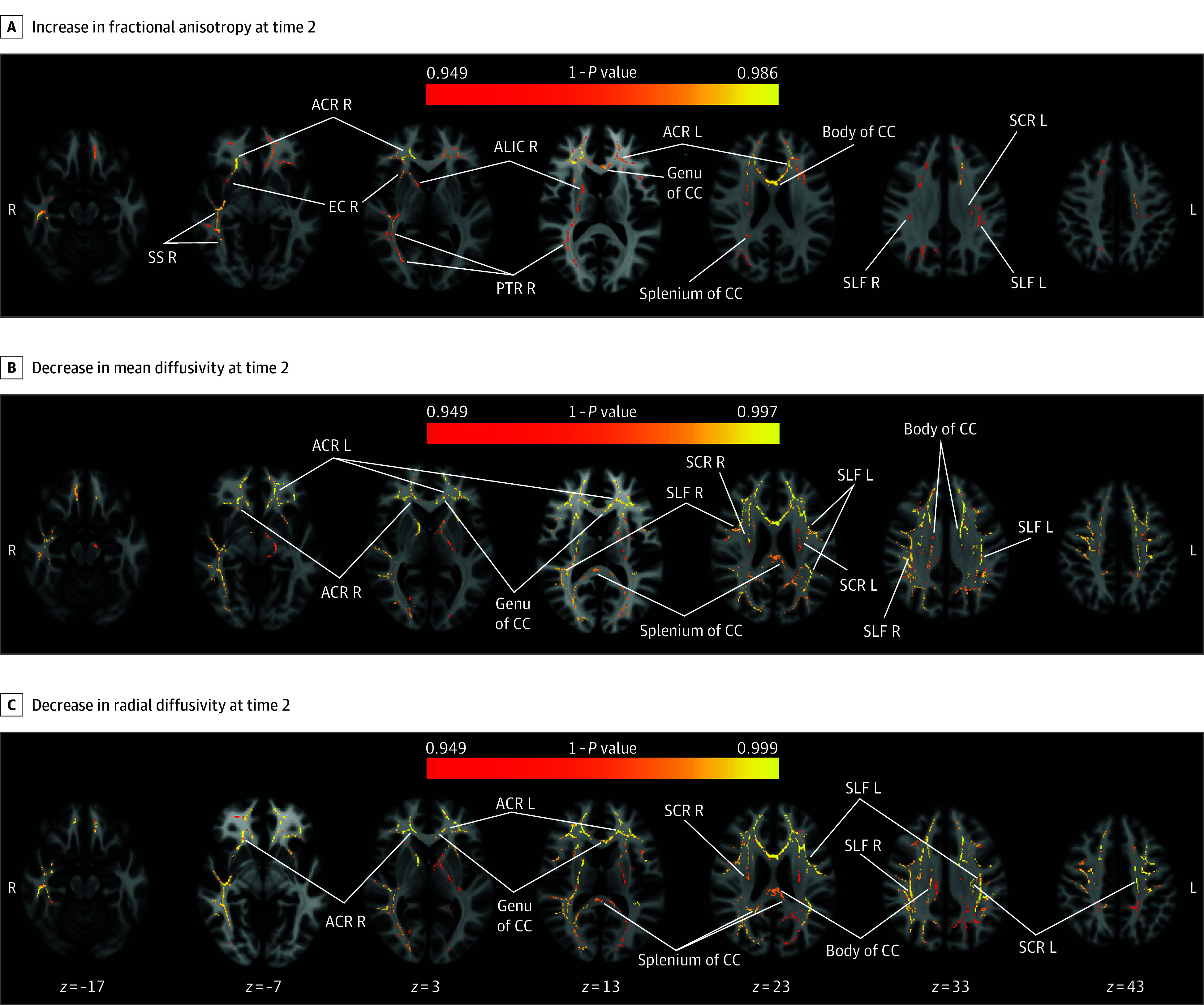
Group-Specific Whole-Brain Differences of Fractional Anisotropy and Mean and Radial Diffusivities Between 2 Magnetic Resonance Imaging (MRI) Scans During Treatment Thresholded maps of significant voxels (1 − *P* > 0.949, Threshold-Free Cluster Enhancement corrected) where the change between baseline and follow-up MRIs differed between healthy controls and individuals with heroin use disorder (HUD). The first row shows significant regions where HUD had higher fractional anisotropy at follow-up vs baseline MRI compared with the control group. The second and third rows show significant voxels where HUD showed decreases in mean and radial diffusivity, respectively, at follow-up vs baseline MRI as compared with the control group. See eTable in Supplement 1 for clustered results. ACR indicates anterior corona radiata; ALIC, anterior limb of the internal capsule; CC, corpus callosum; EC, external capsule; L, left; PTR, posterior thalamic radiation; R, right; SCR, superior corona radiata; SLF, superior longitudinal fasciculus; SS, sagittal stratum.

The diffusion metrics were significantly different between the follow-up and baseline MRIs of individuals with HUD and control in several voxels (Figure 1). Voxelwise group differences analysis with independent groups *t* tests found individuals with HUD to have a significantly greater effects in fractional anisotropy (1 − *P* = 0.949-0.986; individuals with HUD greater increase than control), mean diffusivity (1 − *P* = 0.949-0.997; individuals with HUD greater decrease than control), and radial diffusivity (1 − *P* = 0.949-0.999; individuals with HUD greater decrease than control). No significant effects were found for axial diffusivity. Increased fractional anisotropy in individuals with HUD was localized to parts of the genu, body, and splenium of the corpus callosum as well as in bilateral anterior corona radiata, left superior corona radiata, right anterior limb of internal capsule, right posterior thalamic radiation, bilateral superior longitudinal fasciculus, right sagittal stratum, and right external capsule. Voxels showing decreased mean and radial diffusivity in individuals with HUD were more widespread in voxel count, although still found primarily in the same regions. Specific clustered results and localizations are summarized in the eTable in Supplement 1.

**Figure 2.**
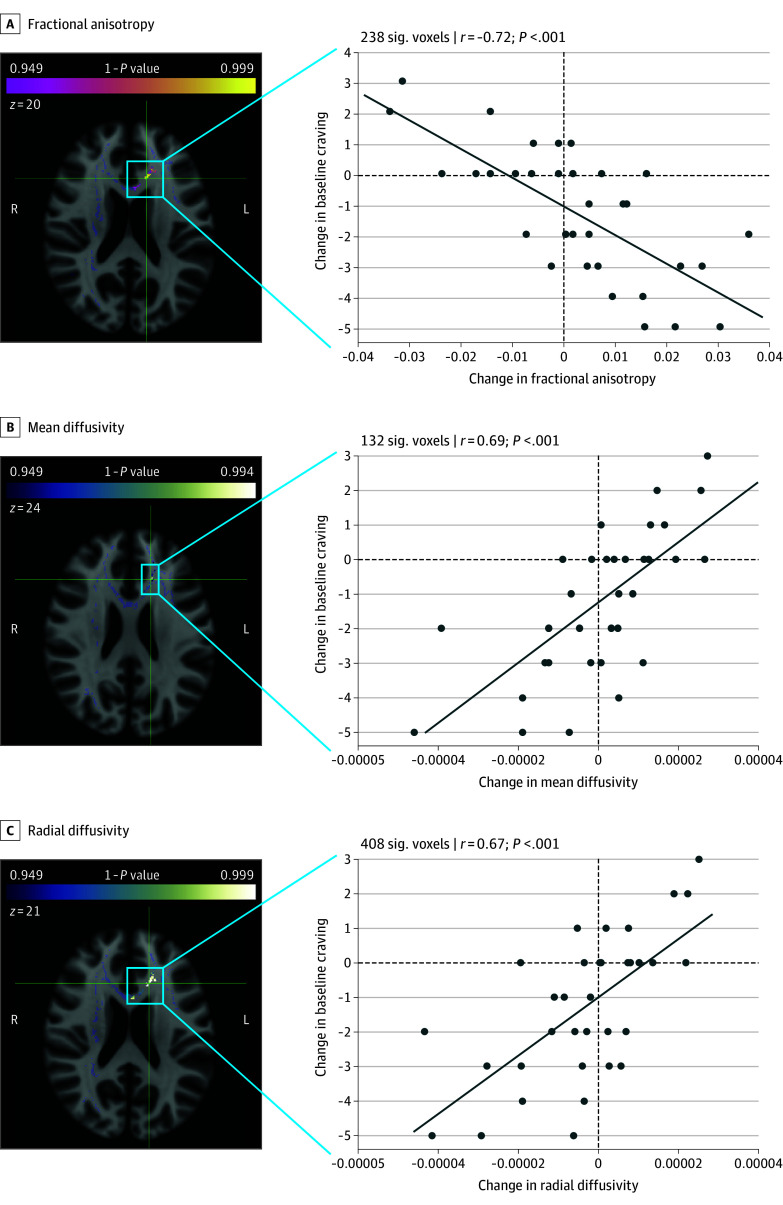
Voxelwise Correlations Between Changes in Diffusion Metrics and Baseline Craving in Individuals With Heroin Use Disorder (HUD) Voxels where the increased fractional anisotropy and decreased mean and radial diffusivity at follow-up magnetic resonance imaging significantly correlated with a reduction of baseline craving. This analysis used the mask of fractional anisotropy recovery shown in Figure 1 (gray). These results were observed mainly in the genu and body of the corpus callosum and the left anterior corona radiata. Green crosshairs indicate the peak voxel location for the correlation with each of the 3 metrics (Montreal Neurological Institute coordinates, fractional anisotropy: x = −13, y = 23, z = 20; mean diffusivity: x = −16, y = 26, z = 24; radial diffusivity: x = −15, y = 24, z = 21).

Voxelwise correlation analyses showed significant correlations (1 − *P* > .949) in the between-scan changes in fractional anisotropy, mean diffusivity, and radial diffusivity, with the change in baseline craving in voxel clusters located in the genu and body of the corpus callosum and the left anterior corona radiata (Figure 2). To assess effect sizes, changes in fractional anisotropy (r = −0.72, *P* < .001, slope SE = 9.0 × 10^−4^), mean diffusivity (r = 0.69, *P* < .001, slope SE = 1.25 × 10^−6^), and radial diffusivity (r = 0.67, *P* < .001, slope SE = 1.16 × 10^−6^) values were averaged across voxels that showed significant correlations with craving in the voxelwise analysis, revealing a negative fractional anisotropy correlation with change in baseline craving and positive correlations for changes in mean and radial diffusivity. Correlations between the white matter metrics and changes in scene-induced craving values did not yield significant results. Similarly, no correlations between the white matter metrics changes in values with the other cue-induced craving measures, therapy attendance, and self-reported methadone and buprenorphine dosages reached significance (eAppendix in Supplement 1).

## Discussion

This study aimed to investigate the white matter changes with inpatient treatment in individuals with HUD undergoing MAT, and their potential associations with changes in baseline and cue-induced craving measures and behavioral clinical measures including mood and affect, sleep, and stress. Our main findings demonstrate that neuroanatomical impairments in distributed frontal white matter tracks in individuals with HUD undergo appreciable change characterized by increased fractional anisotropy and decreased mean and radial diffusivity, indicative of recovery processes. Our findings also show that recovery of white matter microstructure, specifically in the genu and body of the corpus callosum and bilateral anterior corona radiata, correlated with reductions in baseline drug craving.

Multiple studies show white matter impairments across various drugs of abuse including heroin,^15,16,17,18,19,21^ cocaine,^39,40,41,42^ alcohol,^43,44^ nicotine,^45,46,47^ and cannabis.^48^ Cross-sectional studies have suggested a correlation between abstinence and potential white matter recovery, including our prior study in cocaine use disorder in which, compared with active users, abstainers (representing a group with longer abstinence durations and/or less chronicity of use) showed limited impairments compared with control participants.^14^ Corroborating results showed less white matter impairments in long-term drug abstainers (negative urine status for any drugs) compared with individuals with HUD undergoing MAT,^49^ and in heroin abstainers compared with individuals who relapsed to heroin use.^17^

Although limited in number, repeated within-participant studies corroborate these cross-sectional studies.^21^ Tractography studies using atlas-based analyses showed increased fractional anisotropy values in fronto-striatal circuits and nucleus accumbens fiber tracts after 8 vs 2 months of abstinence in heroin users (without MAT).^22,23^ The current study bolstered the fractional anisotropy increase in these networks during treatment, extending results to other diffusion metrics (mean and radial diffusivity) and showing more widespread patterns than previously observed. These broad white matter changes could stem from the relatively enriched services provided to these individuals with HUD (all enrolled in an intensive inpatient biopsychosocial support program with MAT). Taken together, this pattern is consistent with normalization and/or regeneration of axonal processes and remyelination^50,51,52,53,54,55^ in specific white matter tracts with inpatient MAT in individuals with HUD. Noteworthy potential mechanisms include chronic opiate effects (and their change with treatment) on neuroinflammation mediated by microglia^56,57^ and gene expression in oligodendrocytes.^58,59,60,61,62^

The clinical relevance of these results also derives from the significant association of the white matter changes with decreases in baseline craving during treatment. Based on the localization of significant voxels, encompassing potential corticocortical, corticothalamic, and corticolimbic circuits in the left anterior corona radiata and parts of the genu and body of the corpus callosum, these white matter changes could relate to the recovery of higher-order cognitive functions including emotional regulation and top-down executive control.^63,64^ Our findings suggest that more efficient communication in these areas could contribute to reduction in baseline, although not stimulus or cue-induced, craving.^65^

### Limitations

Our results should be interpreted in light of several limitations. First, replication in a larger independent sample is necessary as participants in this study were also included in previous cross-sectional reports.^14,66^ Larger studies are also needed to examine effects on white matter microstructure of individual differences related to sex and gender, treatment-seeking status, and medication (eg, methadone or buprenorphine) during recovery. Although we found no significant association between baseline self-reported methadone or buprenorphine dosages and our dependent measures, more rigorous assessments of recovery effects related to MAT type and dosage are also necessary. Future studies using a within-participant longitudinal design starting immediately upon entering into treatment, or a comparison group of outpatient or non–medication-assisted inpatient individuals with HUD, can also help to provide a better understanding of comparative white matter dynamics over longer time courses and with different treatments. Finally, the model on which TBSS analyses rely is known to lack specificity in areas with complex white matter fiber architecture (including crossing fibers),^67^ precluding the observation of potentially relevant circuitry especially in subcortical white matter regions.

## Conclusions

To our knowledge, this is the first study to investigate whole-brain white matter changes with MAT in individuals with HUD. Our findings demonstrated increased fractional anisotropy and decreased mean and radial diffusivity in fronto-striatal white matter tracts after approximately 15 weeks in MAT in individuals with HUD, consistent with normalization and/or regeneration of white matter microstructure. The association of white matter indices of recovery with the decrease of baseline craving at follow-up further suggests cognitive or motivational improvements with abstinence and treatment in individuals with HUD, which may contribute to longer-term abstinence and relapse prevention.

## Notes

Supplement 1.
eMethods
**eAppendix.** Supplementary Results **eTable.** Clustered Between-Group White Matter Differences on Change of Diffusion Metrics Between MRI 1 and MRI 2

Supplement 2.
Data Sharing Statement

## References

[1] Center for Disease Control and Prevention. Understanding the Opioid Overdose Epidemic. Published August 8, 2023. Accessed August 16, 2023. https://www.cdc.gov/opioids/basics/epidemic.html

[2] Smyth BP, Barry J, Keenan E, Ducray K. Lapse and relapse following inpatient treatment of opiate dependence. Ir Med J. 2010;103(6):176-179. 20669601

[3] McLellan AT, Lewis DC, O’Brien CP, Kleber HD. Drug dependence, a chronic medical illness: implications for treatment, insurance, and outcomes evaluation. JAMA. 2000;284(13):1689-1695. 11015800 10.1001/jama.284.13.1689

[4] Volkow ND, Fowler JS. Addiction, a disease of compulsion and drive: involvement of the orbitofrontal cortex. Cereb Cortex. 2000;10(3):318-325. 10731226 10.1093/cercor/10.3.318

[5] Kadam M, Sinha A, Nimkar S, Matcheswalla Y, De Sousa A. A comparative study of factors associated with relapse in alcohol dependence and opioid dependence. Indian J Psychol Med. 2017;39(5):627-633. 29200559 10.4103/IJPSYM.IJPSYM_356_17 PMC5688890

[6] Vafaie N, Kober H. Association of drug cues and craving with drug use and relapse: a systematic review and meta-analysis. JAMA Psychiatry. 2022;79(7):641-650. 35648415 10.1001/jamapsychiatry.2022.1240 PMC9161117

[7] Ceceli AO, Bradberry CW, Goldstein RZ. The neurobiology of drug addiction: cross-species insights into the dysfunction and recovery of the prefrontal cortex. Neuropsychopharmacology. 2022;47(1):276-291. 34408275 10.1038/s41386-021-01153-9 PMC8617203

[8] Zilverstand A, Huang AS, Alia-Klein N, Goldstein RZ. Neuroimaging impaired response inhibition and salience attribution in human drug addiction: a systematic review. Neuron. 2018;98(5):886-903. 29879391 10.1016/j.neuron.2018.03.048 PMC5995133

[9] Huang Y, Ceceli AO, Kronberg G, . Association of cortico-striatal engagement during cue reactivity, reappraisal, and savoring of drug and non-drug stimuli with craving in heroin addiction. Am J Pyschiatry. 2023;181(2):20220759. 10.1176/appi.ajp.20220759 PMC11034831 37434487

[10] Liu S, Wang S, Zhang M, . Brain responses to drug cues predict craving changes in abstinent heroin users: a preliminary study. Neuroimage. 2021;237:118169. 34000396 10.1016/j.neuroimage.2021.118169

[11] Moeller SJ, Paulus MP. Toward biomarkers of the addicted human brain: using neuroimaging to predict relapse and sustained abstinence in substance use disorder. Prog Neuropsychopharmacol Biol Psychiatry. 2018;80(Pt B):143-154. 28322982 10.1016/j.pnpbp.2017.03.003 PMC5603350

[12] Yip SW, Scheinost D, Potenza MN, Carroll KM. Connectome-based prediction of cocaine abstinence. Am J Psychiatry. 2019;176(2):156-164. 30606049 10.1176/appi.ajp.2018.17101147 PMC6481181

[13] Zilverstand A, Parvaz MA, Moeller SJ, . Whole-brain resting-state connectivity underlying impaired inhibitory control during early versus longer-term abstinence in cocaine addiction. Mol Psychiatry. 2023;28(8):3355-3364. 37528227 10.1038/s41380-023-02199-5 PMC10731999

[14] Gaudreault PO, King SG, Malaker P, Alia-Klein N, Goldstein RZ. Whole-brain white matter abnormalities in human cocaine and heroin use disorders: association with craving, recency, and cumulative use. Molecular Psychiatry. 2023;28:780-791. doi:10.1038/s41380-022-01833-y 36369361 PMC9911401

[15] Bora E, Yücel M, Fornito A, . White matter microstructure in opiate addiction. Addict Biol. 2012;17(1):141-148. 21070508 10.1111/j.1369-1600.2010.00266.x

[16] Li W, Li Q, Zhu J, . White matter impairment in chronic heroin dependence: a quantitative DTI study. Brain Res. 2013;1531:58-64. 23895765 10.1016/j.brainres.2013.07.036

[17] Li W, Zhu J, Li Q, . Brain white matter integrity in heroin addicts during methadone maintenance treatment is related to relapse propensity. Brain Behav. 2016;6(2):e00436. 27110449 10.1002/brb3.436 PMC4834937

[18] Liu H, Li L, Hao Y, . Disrupted white matter integrity in heroin dependence: a controlled study utilizing diffusion tensor imaging. Am J Drug Alcohol Abuse. 2008;34(5):562-575. 18720268 10.1080/00952990802295238

[19] Wollman SC, Alhassoon OM, Stern MJ, . White matter abnormalities in long-term heroin users: a preliminary neuroimaging meta-analysis. Am J Drug Alcohol Abuse. 2015;41(2):133-138. 25664621 10.3109/00952990.2014.985829

[20] Parvaz MA, Rabin RA, Adams F, Goldstein RZ. Structural and functional brain recovery in individuals with substance use disorders during abstinence: a review of longitudinal neuroimaging studies. Drug Alcohol Depend. 2022;232:109319. 35077955 10.1016/j.drugalcdep.2022.109319 PMC8885813

[21] Wang X, Yu R, Zhou X, . Reversible brain white matter microstructure changes in heroin addicts: a longitudinal study. Addict Biol. 2013;18(4):727-728. 21762286 10.1111/j.1369-1600.2011.00316.x

[22] Lu L, Yang W, Zhang X, . Potential brain recovery of frontostriatal circuits in heroin users after prolonged abstinence: a preliminary study. J Psychiatr Res. 2022;152:326-334. 35785575 10.1016/j.jpsychires.2022.06.036

[23] Lu L, Yang W, Zhao D, . Brain recovery of the NAc fibers and prediction of craving changes in person with heroin addiction: a longitudinal study. Drug Alcohol Depend. 2023;243:109749. 36565569 10.1016/j.drugalcdep.2022.109749

[24] Sheehan DV, Lecrubier Y, Sheehan KH, . The Mini-International Neuropsychiatric Interview (MINI): the development and validation of a structured diagnostic psychiatric interview for DSM-IV and ICD-10. J Clin Psychiatry. 1998;59(suppl 20):22-33. 9881538

[25] McLellan AT, Kushner H, Metzger D, . The fifth edition of the addiction severity index. J Substance Abuse Treatment. 1992;9(3):199-213. 10.1016/0740-5472(92)90062-s 1334156

[26] Gossop M. The development of a short opiate withdrawal scale (SOWS). Addict Behav. 1990;15(5):487-490. 2248123 10.1016/0306-4603(90)90036-w

[27] Tiffany ST, Singleton E, Haertzen CA, Henningfield JE. The development of a cocaine craving questionnaire. Drug Alcohol Depend. 1993;34(1):19-28. 8174499 10.1016/0376-8716(93)90042-o

[28] Heinz AJ, Epstein DH, Schroeder JR, Singleton EG, Heishman SJ, Preston KL. Heroin and cocaine craving and use during treatment: measurement validation and potential relationships. J Subst Abuse Treat. 2006;31(4):355-364. 17084789 10.1016/j.jsat.2006.05.009

[29] Gossop M, Griffiths P, Powis B, Strang J. Severity of dependence and route of administration of heroin, cocaine and amphetamines. Br J Addict. 1992;87(11):1527-1536. 1458032 10.1111/j.1360-0443.1992.tb02660.x

[30] Heatherton TF, Kozlowski LT, Frecker RC, Fagerström KO. The Fagerström Test for Nicotine Dependence: a revision of the Fagerström Tolerance Questionnaire. Br J Addict. 1991;86(9):1119-1127. 1932883 10.1111/j.1360-0443.1991.tb01879.x

[31] Kronberg G, Ceceli AO, Huang Y, . Naturalistic drug cue reactivity in heroin use disorder: orbitofrontal synchronization as a marker of craving and recovery. medRxiv. Preprint published online April 19, 2024:2023.11.02.23297937.

[32] Tournier JD. Diffusion MRI in the brain—theory and concepts. Prog Nucl Magn Reson Spectrosc. 2019;112-113:1-16. 31481155 10.1016/j.pnmrs.2019.03.001

[33] Smith SM, Jenkinson M, Woolrich MW, . Advances in functional and structural MR image analysis and implementation as FSL. Neuroimage. 2004;23(suppl 1):S208-S219. 15501092 10.1016/j.neuroimage.2004.07.051

[34] Smith SM, Jenkinson M, Johansen-Berg H, . Tract-based spatial statistics: voxelwise analysis of multi-subject diffusion data. Neuroimage. 2006;31(4):1487-1505. 16624579 10.1016/j.neuroimage.2006.02.024

[35] Winkler AM, Ridgway GR, Webster MA, Smith SM, Nichols TE. Permutation inference for the general linear model. Neuroimage. 2014;92(100):381-397. 24530839 10.1016/j.neuroimage.2014.01.060 PMC4010955

[36] Fischl B. FreeSurfer. Neuroimage. 2012;62(2):774-781. 22248573 10.1016/j.neuroimage.2012.01.021 PMC3685476

[37] Smith SM, Nichols TE. Threshold-free cluster enhancement: addressing problems of smoothing, threshold dependence and localisation in cluster inference. Neuroimage. 2009;44(1):83-98. 18501637 10.1016/j.neuroimage.2008.03.061

[38] Mori S, Oishi K, Jiang H, . Stereotaxic white matter atlas based on diffusion tensor imaging in an ICBM template. Neuroimage. 2008;40(2):570-582. 18255316 10.1016/j.neuroimage.2007.12.035 PMC2478641

[39] He Q, Li D, Turel O, Bechara A, Hser YI. White matter integrity alternations associated with cocaine dependence and long-term abstinence: Preliminary findings. Behav Brain Res. 2020;379:112388. 31783090 10.1016/j.bbr.2019.112388

[40] Ottino-González J, Uhlmann A, Hahn S, . White matter microstructure differences in individuals with dependence on cocaine, methamphetamine, and nicotine: findings from the ENIGMA-Addiction working group. Drug Alcohol Depend. 2022;230:109185. 34861493 10.1016/j.drugalcdep.2021.109185 PMC8952409

[41] Suchting R, Beard CL, Schmitz JM, . A meta-analysis of tract-based spatial statistics studies examining white matter integrity in cocaine use disorder. Addict Biol. 2021;26(2):e12902. 32267062 10.1111/adb.12902 PMC7541563

[42] Tondo LP, Viola TW, Fries GR, . White matter deficits in cocaine use disorder: convergent evidence from in vivo diffusion tensor imaging and ex vivo proteomic analysis. Transl Psychiatry. 2021;11(1):252. 33911068 10.1038/s41398-021-01367-x PMC8081729

[43] Fortier CB, Leritz EC, Salat DH, . Widespread effects of alcohol on white matter microstructure. Alcohol Clin Exp Res. 2014;38(12):2925-2933. 25406797 10.1111/acer.12568 PMC4293208

[44] Jansen JM, van Holst RJ, van den Brink W, Veltman DJ, Caan MWA, Goudriaan AE. Brain function during cognitive flexibility and white matter integrity in alcohol-dependent patients, problematic drinkers and healthy controls. Addict Biol. 2015;20(5):979-989. 25477246 10.1111/adb.12199

[45] Baeza-Loya S, Velasquez KM, Molfese DL, . Anterior cingulum white matter is altered in tobacco smokers. Am J Addict. 2016;25(3):210-214. 27001211 10.1111/ajad.12362 PMC11684460

[46] Lin F, Wu G, Zhu L, Lei H. Heavy smokers show abnormal microstructural integrity in the anterior corpus callosum: a diffusion tensor imaging study with tract-based spatial statistics. Drug Alcohol Depend. 2013;129(1-2):82-87. 23062873 10.1016/j.drugalcdep.2012.09.013

[47] Savjani RR, Velasquez KM, Thompson-Lake DGY, . Characterizing white matter changes in cigarette smokers via diffusion tensor imaging. Drug Alcohol Depend. 2014;145:134-142. 25457737 10.1016/j.drugalcdep.2014.10.006

[48] Becker MP, Collins PF, Lim KO, Muetzel RL, Luciana M. Longitudinal changes in white matter microstructure after heavy cannabis use. Dev Cogn Neurosci. 2015;16:23-35. 26602958 10.1016/j.dcn.2015.10.004 PMC4691379

[49] Wang Y, Li W, Li Q, Yang W, Zhu J, Wang W. White matter impairment in heroin addicts undergoing methadone maintenance treatment and prolonged abstinence: a preliminary DTI study. Neurosci Lett. 2011;494(1):49-53. 21362458 10.1016/j.neulet.2011.02.053

[50] Le Bihan D. Looking into the functional architecture of the brain with diffusion MRI. Nat Rev Neurosci. 2003;4(6):469-480. 12778119 10.1038/nrn1119

[51] Moore EE, Hohman TJ, Badami FS, . Neurofilament relates to white matter microstructure in older adults. Neurobiol Aging. 2018;70:233-241. 30036759 10.1016/j.neurobiolaging.2018.06.023 PMC6119102

[52] Pierpaoli C, Barnett A, Pajevic S, . Water diffusion changes in Wallerian degeneration and their dependence on white matter architecture. Neuroimage. 2001;13(6 Pt 1):1174-1185. 11352623 10.1006/nimg.2001.0765

[53] Pierpaoli C, Basser PJ. Toward a quantitative assessment of diffusion anisotropy. Magn Reson Med. 1996;36(6):893-906. 8946355 10.1002/mrm.1910360612

[54] Wheeler-Kingshott CAM, Cercignani M. About “axial” and “radial” diffusivities. Magn Reson Med. 2009;61(5):1255-1260. 19253405 10.1002/mrm.21965

[55] Winklewski PJ, Sabisz A, Naumczyk P, Jodzio K, Szurowska E, Szarmach A. Understanding the physiopathology behind axial and radial diffusivity changes—what do we know? Front Neurol. 2018;9:92. 29535676 10.3389/fneur.2018.00092 PMC5835085

[56] Maduna T, Audouard E, Dembélé D, . Microglia express mu opioid receptor: insights from transcriptomics and fluorescent reporter mice. Front Psychiatry. 2019;9:726. 30662412 10.3389/fpsyt.2018.00726 PMC6328486

[57] Reiss D, Maduna T, Maurin H, Audouard E, Gaveriaux-Ruff C. Mu opioid receptor in microglia contributes to morphine analgesic tolerance, hyperalgesia, and withdrawal in mice. J Neurosci Res. 2022;100(1):203-219. 32253777 10.1002/jnr.24626

[58] Belachew S, Rogister B, Rigo JM, Malgrange B, Moonen G. Neurotransmitter-mediated regulation of CNS myelination: a review. Acta Neurol Belg. 1999;99(1):21-31. 10218089

[59] Fan R, Schrott LM, Arnold T, . Chronic oxycodone induces axonal degeneration in rat brain. BMC Neurosci. 2018;19(1):15. 29571287 10.1186/s12868-018-0417-0 PMC5865283

[60] Johnson SW, North RA. Opioids excite dopamine neurons by hyperpolarization of local interneurons. J Neurosci. 1992;12(2):483-488. 1346804 10.1523/JNEUROSCI.12-02-00483.1992 PMC6575608

[61] Zhang Y, Zhang H, Wang L, . Quetiapine enhances oligodendrocyte regeneration and myelin repair after cuprizone-induced demyelination. Schizophr Res. 2012;138(1):8-17. 22555017 10.1016/j.schres.2012.04.006

[62] Reiner BC, Zhang Y, Stein LM, . Single nucleus transcriptomic analysis of rat nucleus accumbens reveals cell type-specific patterns of gene expression associated with volitional morphine intake. Transl Psychiatry. 2022;12(1):374. 36075888 10.1038/s41398-022-02135-1 PMC9458645

[63] Sanjuan PM, Thoma R, Claus ED, Mays N, Caprihan A. Reduced white matter integrity in the cingulum and anterior corona radiata in posttraumatic stress disorder in male combat veterans: a diffusion tensor imaging study. Psychiatry Res. 2013;214(3):260-268. 24074963 10.1016/j.pscychresns.2013.09.002 PMC3988979

[64] Wakana S, Jiang H, Nagae-Poetscher LM, van Zijl PCM, Mori S. Fiber tract-based atlas of human white matter anatomy. Radiology. 2004;230(1):77-87. 14645885 10.1148/radiol.2301021640

[65] Etkin A, Egner T, Kalisch R. Emotional processing in anterior cingulate and medial prefrontal cortex. Trends Cogn Sci. 2011;15(2):85-93. 21167765 10.1016/j.tics.2010.11.004 PMC3035157

[66] King SG, Gaudreault PO, Malaker P, . Prefrontal-habenular microstructural impairments in human cocaine and heroin addiction. Neuron. 2022;110(22):3820-3832.e4. 36206758 10.1016/j.neuron.2022.09.011 PMC9671835

[67] Jeurissen B, Leemans A, Tournier JD, Jones DK, Sijbers J. Investigating the prevalence of complex fiber configurations in white matter tissue with diffusion magnetic resonance imaging. Hum Brain Mapp. 2013;34(11):2747-2766. 22611035 10.1002/hbm.22099 PMC6870534

