# Supplementary material for: Frontal White Matter Changes and Craving Recovery in Inpatients With Heroin Use Disorder
Source: JAMA Netw Open. 2024 Dec 18;7(12):e2451678. doi: 10.1001/jamanetworkopen.2024.51678 (PMC11656271; doi:10.1001/jamanetworkopen.2024.51678)
Supplement: Supplement 2. — Data Sharing Statement [file jamanetwopen-e2451678-s002.pdf]

Data Sharing Statement

Gaudreault. Frontal White Matter Changes and Craving Recovery in Inpatients With Heroin Use Disorder. *JAMA Netw Open*. Published December 18, 2024.
doi:10.1001/jamanetworkopen.2024.51678

Data
Data available: Yes
Data types: Deidentified participant data, Data dictionary
How to access data: rita.goldstein@mssm.edu
When available: With publication

Supporting Documents
Document types: None

Additional Information
Who can access the data: rita.goldstein@mssm.edu
Types of analyses: Any purpose
Mechanisms of data availability: After a signed data access agreement
